# Trans-axillary single port insufflation technique-assisted endoscopic surgery for breast diseases: Clinic experience, cosmetic outcome and oncologic result

**DOI:** 10.3389/fonc.2023.1157545

**Published:** 2023-03-29

**Authors:** Xuefei Wang, Xin Wan, Lifang Li, Xu Liu, Ran Meng, Xiaohu Sun, Chunhua Xiao

**Affiliations:** ^1^ Tianjin Medical University Cancer Institute and Hospital, National Clinical Research Center for Cancer Medical, Tianjin, China; ^2^ Tianjin Medical University Cancer Institute and Hospital, Key Laboratory of Cancer Prevention and Therapy, Tianjin, China; ^3^ The First Surgical Department of Breast Cancer, Tianjin’s Clinical Research Center for Cancer, Tianjin, China; ^4^ Key Laboratory of Breast Cancer Prevention and Therapy, Tianjin Medical University, Ministry of Education, Tianjin, China; ^5^ Graduate School, Tianjin Medical University, Tianjin, China

**Keywords:** breast cancer, endoscopic surgery, single incision, cosmetic outcome, oncologic safety

## Abstract

**Purpose:**

With an increasing demand for postoperative cosmetic effects in breast diseases, the single port by trans-axillary incision and air-inflation system, which provided better space and spared the assistant the effort of retraction, is widely used in clinic surgical treatment for multiple breast diseases.

**Methods:**

According to inclusion and exclusion criteria, patients who underwent trans-axillary single-incision surgery at Tianjin Medical University Cancer Hospital between December 2020 and July 2022 were included in the study. We collected and analyzed data on age, fertility history, ultrasound grade, clinical stage, pathological results, oncological prognosis, patient-centered cosmetic outcome, etc.

**Results:**

A total of 115 cases were included, of which 33 patients with benign disease underwent mass resection, 68 patients with malignant tumors underwent mastectomy. 10 patients had a special type of breast lesion. A mastectomy was performed in 4 patients with male mammary gland development. Of the 115 cases, the maximum mass diameter was 3.00 ± 1.644 (0.6–8.5) cm. Blood loss during surgery was 85.77 ± 50.342 (10-200) ml. The surgery took 131.84 ± 59.332 (30-280) minutes to complete. The patient spent a total of 5.05 ± 2.305 (2-18) days in the hospital. And the length of surgical incision in all patients was 3.83 ± 0.884 (3-8) cm. All patients were very satisfied with the appearance of their breasts after dressing. 94.78% of patients were satisfied with the position of the incision.

**Conclusion:**

Through this study, we believe that in benign breast diseases and malignant breast tumors, trans-axillary single port insufflation technique-assisted endoscopic surgery has oncological safety and an aesthetic effect for most people with breast diseases.

## Introduction

In recent decades, with advances in medicine and innovations in medical technology, the methods of radical mastectomy have been updated and improved as needed. Nipple-sparing mastectomy (NSM) with preservation of the flap and nipple–areola complex (NAC) was widely used in breast cancer surgery throughout the world in patients with no sign of nippleareola invasion. It provides oncologic safety, improved reconstructive outcomes and patient satisfaction (1–4). In fact, the traditional incisions of the NSM, namely the radial incision around the nipple or the incision under the inframammary fold, provide a better and more comfortable access for the placement of implants or expanders during surgery (5, 6). However, it should be understood that both the radial and inframammary fold incisions leave visible surgical scars on the breast surface, which obviously has a negative impact on the breast reconstruction process (6, 7). Furthermore, in terms of aesthetic outcomes, the traditional radial and inframammary incision scars leave a permanent, visible scar on the breast mound, which is an immediate and lifelong mark of breast surgery in both mastectomy and breast reconstruction (5, 7, 8). So people were beginning to realize that the traditional incision of NSM not only affects the outcome of breast reconstruction but also has a psychological impact on the patient.

The ever-increasing need to further improve aesthetic outcomes in the surgical treatment of the breast has led to new NSM surgical innovations such as endoscopic-assisted nipple-sparing mastectomy (E-NSM) (9–11) and robotic nipple-sparing mastectomy (R-NSM) (12–14), which are emerging and increasingly being used in the surgical treatment of breast cancer (9, 15–17). Since the application of endoscopic techniques was first reported in 1998, a growing number of reports have shown that endoscopic techniques are a safe oncological procedure (4, 18). In conventional E-NSM, two to three incisions are made in the axilla and around the NAC (9, 19), and the axillary incision is sometimes extended beyond 5 cm to remove breast tissue, which is often out of line with the skin crease and line orientation (10), these surgical incisions and inconsistencies in the direction of the skin line often result in significant post-operative scarring, which can seriously affect the appearance. In addition, the incisions around NAC were associated with the incidence of NAC ischemia and necrosis in this surgical approach, and conventional E-NSM surgery is not significantly better in this respect compared to conventional NSM (7–9, 19–21). As reported, E-NSM had the advantages of a shorter operation time, lower cost, and fewer instruments required compared to R-NSM (22). Therefore, the E-MSN with a single axillary incision after improving has gradually replaced the traditional surgery with multiple incisions under the axilla and around the NAC.

The transaxillary nipple-sparing mastectomy using a simplified endoscopic approach is an original method based on the traditional NSM with several improvements. The modified transaxillary NSM solves, to some extent, the problems of scar healing after conventional mastectomy, excessive scar growth and the effect of scar contracture on post-operative breast reconstruction (7, 15, 17, 23). Its outcomes have been recognized by patients. The new technical modifications to E-NSM focus on the single axillary incision and the air inflation system has the advantages of not only reducing the incidence of ischemia and necrosis, but also improving the aesthetics of the incision scar healing by shortening the length of the wound and reducing the number of incisions (15, 24). So based on the long-term aesthetic pursuit of the patient, the NSM using a single transaxillary incision and insufflation technique-assisted endoscopic approach has been promoted in clinical practice.

The purpose of this paper is to report our experience with trans-axillary single port insufflation technique-assisted endoscopic surgery for breast diseases and analyze clinic experience, cosmetic outcome and oncologic result in China by focusing on surgery indications and technical refinements.

## Patients and methods

### Patients

The indications for trans-axillary single port insufflation technique-assisted endoscopic surgery, that is, the conditions to be included in patients undergoing this operation, are: body mass index (BMI) less than 30; the breast sizes are below C cup; the lesions are more than 1 cm away from the nipple–areola complex; the lesions are less than 5 cm in diameter in each of the four quadrants; and the patients should be classified as low-risk anesthesia, who have no associated comorbidities, such as cardiovascular and cerebrovascular diseases. And patients with significant NAC involvement, breast cancer with chest wall or skin invasion, breast cancer with extensive axillary lymph node metastasis or locally advanced breast cancer, prior radiation therapy, severe heart disease, renal failure, or liver dysfunction as assessed by the clinician, should be contraindicated for this kind of E-NSM.

During December 2020 and July 2022 in the First Breast Department of Tianjin Medical University Cancer Hospital, we included 115 patients who met the criteria for breast disease-related surgery with a single axillary incision insufflation technique assisted endoscopically, all patients had signed relevant informed consent documents before the surgery. We divided all patients into four groups: the benign lesion group, the malignant tumor group, the special group, and the male patient group. In the malignant tumor group, patients underwent nipple-sparing areola mastectomy or breast-conserving surgery with excising the breast mass. Patients in this special group were those who underwent surgery with recurrent breast diseases, all of them had a previous history of breast surgery, such as breast augmentation, breast mastectomy combined reconstruction, or chemical injection breast augmentation. In order to evaluate the feasibility and safety of this approach, we sorted out and classified some information of the patients, which mainly includes general patient information such as age, reproductive history, previous breast surgery, grading of breast ultrasound, pathological types and stages, molecular subtype, etc. and surgery-related information mainly including tumor size, distance of lesion from nipple, operative program, incision size, duration of operation, intraoperative blood loss, hospital stays, etc. For the evaluation of oncological prognosis, the positive rates of surgical margin involvement, local recurrence rate, distant metastasis, disease-free survival (DFS), and overall survival (OS) were observed. Surgical margin involvement was defined as “ink” on the tumor. Recurrence of breast cancer or an ipsilateral axillary tumor was defined as *in situ* and regional recurrence. The incidence of breast cancer recurrence or death was determined at the last follow-up visit on November 30, 2022.

### Patient based aesthetic evaluation

The aesthetic effect of single-axillary-incision insufflation technique-assisted endoscopic was evaluated by comparing the change of breast appearance between the preoperative and the postoperative. Patients or family members were followed up at 3 months after surgery. Breast-Q is a common scale used to evaluate the effect of breast cancer surgery. Therefore, we developed an aesthetic evaluation scale for axillary single-port inflatable endoscopic breast surgery to evaluate the aesthetic effect of endoscopic breast surgery according to the evaluation item of Breast-Q and the scale used by Lai and his team (15, 16). The scale consisted of 8 questions. The patients or their family members were asked to compare the appearance of the breast preoperative and postoperative, the position of the nipple, the volume symmetry of the bilateral breast, and the questions related to the surgical scar (**Table 1**). The follow-up results were divided into four grades: “very satisfied”, “satisfied”, “general” and “dissatisfied”. Patients who answered “very satisfied” or “satisfied” were defined as satisfied with the results.

### Statistical analyses

In the full text, an independent-samples T test was used for comparisons of differences in continuous variables, and results are reported as mean ± standard deviation (SD). Categorical data were tested by the Chi-square test. A p value < 0.05 was considered to indicate statistical significance. All tests were two-tailed. All statistical analyses were performed using the statistical package SPSS (version 25.0, SPSS, Chicago, IL).

## Technique

The process of trans-axillary single-port insufflation technique-assisted endoscopic surgery that our team used is described below.

### Axillary lymph node dissection

**Figure 1:**
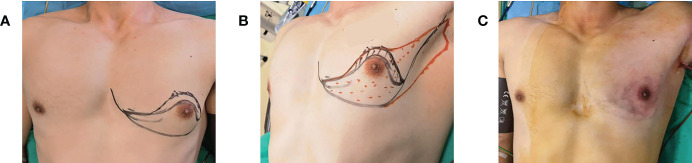
**(A)** Marking the axillary incision and extent of gland removal (frontal view). **(B)** Marking the axillary incision and extent of gland removal (lateral view). **(C)** Symmetrical breast shape and no scarring on the surface after surgery.

**Figure 2:**
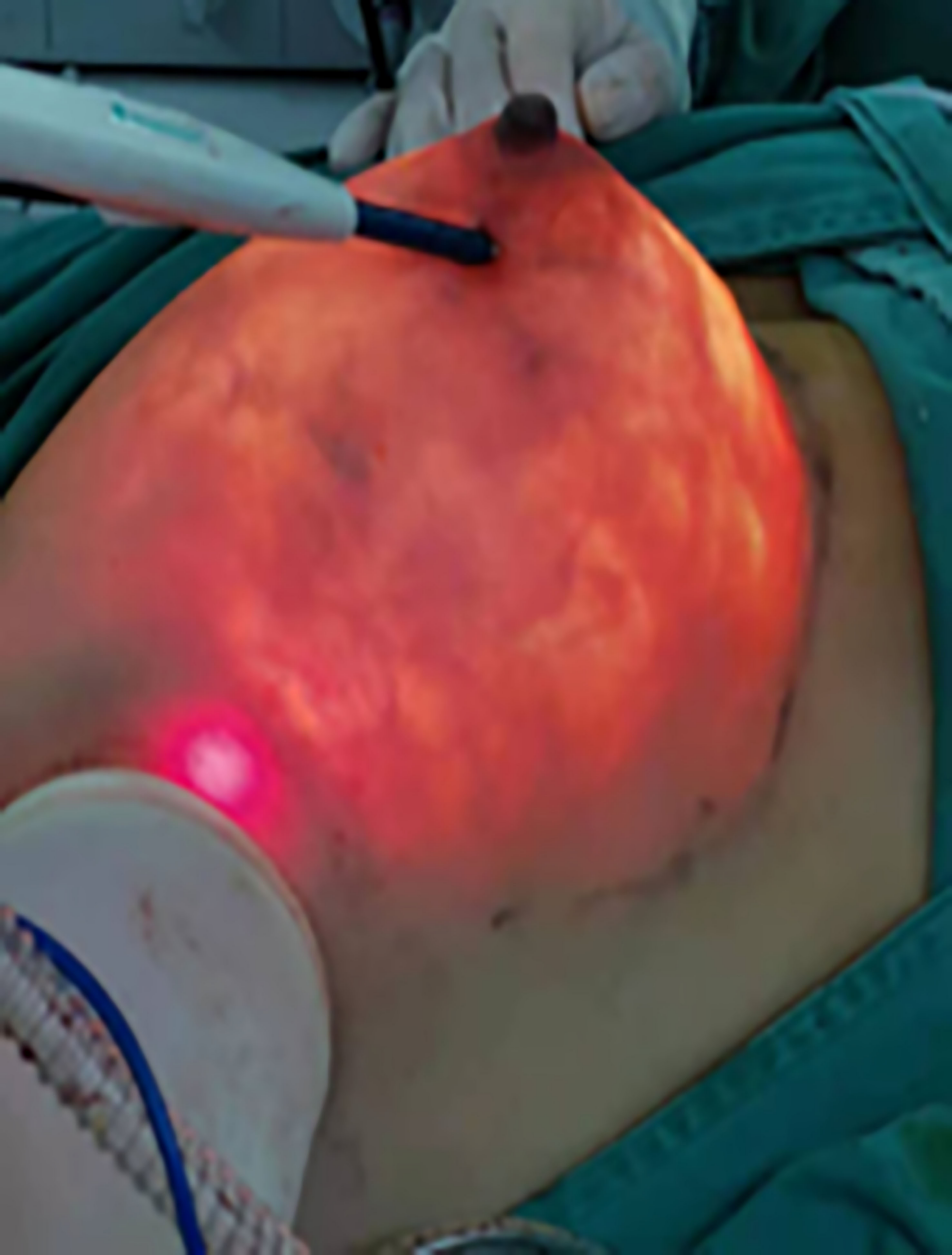
Intraoperative gas build-up to create the space required for the operation.

The free boundary of the areola, the sub-mammary fold, the mass surface projection, and the estimated sentinel lymph node location were marked in the standing position before surgery. After general anesthesia, the patient was placed in a supine position with the upper extremity of the lesion side fixed at 90° abduction to fully expose the incision location for intraoperative manipulation. And then, an oblique axillary incision of approximately 3–5-cm was made in the extra-mammary region. The actual length of the incision depended on the size of the breast to be removed (**Figure 1**). If the preoperative examination indicated the need for sentinel lymph node biopsy (SLNB), methylene blue was injected subcutaneously around the nipple and areola, and colored sentinel lymph nodes were searched according to the preoperative sentinel lymph node surface location. If the SLN was positive, axillary lymph node dissection (ALND), which included the removal of level I and II lymph nodes, is required. A protective sleeve is first placed over the axillary incision, and then carbon dioxide is injected to create an air chamber suitable for this space. The CO2 flow rate was set at 20 L/min to stabilize the chamber pressure in the range of 10-12 mmHg (**Figure 2**). After satisfactory inflation, the axillary fascia is incised horizontally in front of the axillary vein sheath to clearly separate the axillary veins. The axillary lymphatic fatty tissue was removed, and the branches of the chest wall of the axillary vessels are ligated with hemostatic forceps. Meantime, the intercostobrachial nerve, the long thoracic nerve, the thoracodorsal nerve and the subscapular artery should be preserved.

### Total mastectomy and immediate breast reconstruction

**Figure 3:**
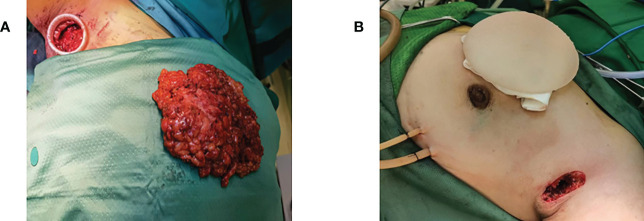
**(A)** Complete removal of the excised gland through the axillary incision after total mastectomy. **(B)** Implant placement *via* axillary incision after total mastectomy.

After SLNB, skin and subcutaneous tissue were separated to fully expose the dissection of the outer margin of the pectoralis major muscle. Then confirm the clear boundary between the pectoral muscle and the breast parenchyma. The posterior breast space and subcutaneous glands completely separated, inward to the sternum, outward to the anterior border of the latissimus dorsi muscle, upward to the subclavicle, and downward to the superior border of the anterior sheath of the rectus abdominal muscle. The extent of a total mastectomy was consistent with a modified radical mastectomy. Taking two separate sub-nipple biopsy specimens from under the NAC was one of the most critical steps, and the intra-operative frozen section was analyzed. If cancer cell invasion was found in the sub-areolar area, the entire NAC had to be removed, and the skin-sparing mastectomy (SSM) was performed instead of the traditional NSM. After completion of the mastectomy, the surgical cavity within the breast was flushed and the operating device is evacuated. The appropriate prosthesis was selected according to the measured volume of the excised gland. The prosthesis is pretreated with an antibiotic soak and gently placed in front of the pectoralis major muscle through an axillary incision. If the patient requires subsequent radiotherapy and it is not appropriate to place the prosthesis, an expander can be placed through the axillary incision. After filling the expander with water based on the size of the breast, the expander injection seat was buried in the subcutaneous layer of the axilla (**Figure 3**). Finally, the incision is closed layer by layer, while drains were placed in the axilla and around the implant or expander.

### Breast-conserving mastectomy

**Figure 4:**
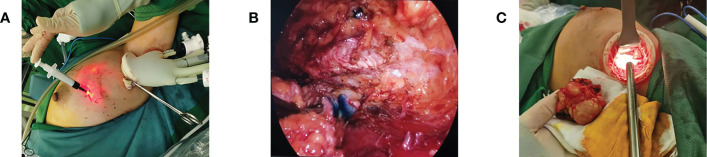
Breast-conserving surgery. **(A)** Preoperative injection of nanocarbon to locate the extent of excision. **(B)** Intraoperative lumpectomy showing the extent of carbon nanostaining. **(C)** Removal of mass *via* axillary incision.

If breast-conserving surgery was performed, the breast tissue is stripped along the posterior space after the gas cavity was established, just like in the mastectomy. According to the physical position identification or methylene blue guidance, the tunnel was separated to the deep surface of the tumor, the fascia and deep glands are excised layer by layer to 1-2 cm outside the edge of the tumor, and then the tumor and surrounding sub glands are completely excised (**Figure 4**). The next step was selected based on the intraoperative frozen pathology results. If the pathological report was negative, adequate irrigation of the incision and strict hemostasis were recommended. After placing the drainage tube, close the incision layer by layer. If the frozen pathology showed the positive tumor margin, the enlarged resection should be performed. When entering the posterior space, it is necessary to try to avoid damaging too much posterior space tissue and normal breast tissue around the tumor, which is conducive to nerve and tissue repair in a short time after the operation.

### Other operations

Another specific type of surgery is prosthetic surgery, such as prosthesis rupture after breast augmentation, prosthesis rupture after breast reconstruction, or chemical injection breast augmentation. We also use the trans-axillary single-port insufflation technique-assisted endoscopic surgery. The key step of the operation is to find the ruptured prosthesis and completely expose it. After taking out the prosthesis capsule completely, rinse surgery field thoroughly with hydrogen peroxide and normal saline. And then choose whether to place a new prosthesis according to the wishes of the patient.

### Postoperative management

The patient was placed in a sedated position for 24 hours after surgery to prevent active bleeding from the surgical area. 24 hours later, the patient was encouraged to get out of bed. The drainage was recorded daily and the color was observed until the drainage turned pale yellow and the daily flow from each drainage bottle was reduced to less than 20ml, then the drainage tube could be removed.

## Result

Of the total 115 patients, 111 (96.52%) were female and 4 (3.48%) were male. Among all female patients, there were 33 (28.70%) patients with benign lesions and 68 (59.13%) patients had malignant tumors. The remaining 10 (8.70%) cases among the female group were patients with recurrent breast diseases who had histories of previous breast surgery. Of the 68 patients with malignancy, 14 (12.17%) received NSM only and 54 (46.96%) underwent mastectomy followed by immediate breast reconstruction. We conducted statistical analysis on the patient information of the benign lesion group (N = 33, 28.70%), the malignant lesion group (N = 68, 59.13%), the special surgery group (N = 10, 8.70%), and the male patient group (N = 4, 3.48%), and also integrated all patient information for a unified analysis. The basic clinical information of all patients is summarized as shown in **Table 2**, the specific pathology and immunohistochemistry information of malignant lesion group shown in **Table 3**, and the surgery-related information is detailed in **Table 1**.

**Table 1:** Patient-oriented outcome report.

Questions Result	Unsatisfied	Fair	Satisfied	Very satisfied	Mean ± standard deviation
1.Postoperative dressed breast appearance satisfaction	0%	0%	88(76.52%)	27(23.48%)	3.23 ± 0.424
2.Postoperative naked breast appearance satisfaction	0%	28(24.35%)	66(57.39%)	21(18.26%)	2.94 ± 0.650
3.Postoperative bilateral breast size satisfaction	0%	27(23.48%)	65(56.52%)	23(20.00%)	2.97 ± 0.658
4.Postoperative bilateral breast symmetry satisfaction	0%	41(35.65%)	50(43.48%)	24(20.87%)	2.85 ± 0.737
5.Postoperative nipple–areola position satisfaction	0%	11(9.57%)	84(73.04%)	20(17.39%)	3.08 ± 0.513
6.Surgical wound position satisfaction	0%	6(5.22%)	41(35.65%)	68(59.13%)	3.54 ± 0.594
7.Scar length satisfaction	0%	15(13.04%)	63(54.78%)	37(32.17%)	3.19 ± 0.645
8.Wound healing satisfaction	0%	6(5.22%)	84(73.04%)	25(21.74%)	3.17 ± 0.492

**Table 2:** Clinical characteristics of all patients.

	Benign(N=33)	Malignancy (N=68)			Special Type(N=10)	Male Breast Cancer (N=4)	All patients(N=115)
		Mastectomy(N=14)	Mastectomy and Immediate Breast Conserving (N=54)	Malignancy(N=68)			
**Age (years/mean)**	25.94 ± 11.034(11-60)	43.79 ± 10.213(30-67)	41.13 ± 5.944(27-55)	41.68 ± 7.120(27-67)	47.40 ± 8.732(33-66)	22.00 ± 5.244(15-28)	36.97 ± 11.737(11-67)
Pregnancy history							
Yes	12(36.36%)	13(92.86%)	52(96.30%)	65(95.59%)	7(70.00%)	/	84(75.68%)
No	21(63.64%)	1(7.14%)	2(3.70%)	3(4.41%)	3(30.00%)	/	27(24.32%)
Menstrual history							
Premenopause	33(100%)	13(92.86%)	53(98.15%)	66(97.06%)	9(90.00%)	/	108(97.30%)
Postmenopause	0(0%)	1(7.14%)	1(1.85%)	2(2.94%)	1(10.00%)	/	3(2.70%)
History of breast surgery							
Yes	3(9.09%)	2(14.28%)	9(16.67%)	11(16.18%)	10(100%)	1(25.00%)	25(21.74%)
No	30(90.91%)	12(85.71%)	45(83.33%)	57(83.32%)	0(0%)	3(75.00%)	90(78.26%)
Location							
Left	15(45.45%)	9(64.29%)	31(57.41%)	40(58.82%)	1(10.00%)	3(75.00%)	59(51.30%)
Right	15(45.45%)	5(35.71%)	23(42.59%)	28(41.18%)	7(70.00%)	1(25.00%)	51(44.35%)
Bilateral	3(9.09%)	0(0%)	0(0%)	0(0%)	2(20.00%)	0(0%)	5(4.35%)
**Tumor Size**	4.13 ± 1.731(1.3-8.5)	2.46 ± 0.970(1.2-4.7)	2.41 ± 1.232(0.6-5.8)	2.42 ± 1.182(0.6-5.8)	4.53 ± 1.808(2.0-6.1)	1.38 ± 0.540(0.8-2.1)	3.00 ± 1.644(0.6-8.5)
BI-RADS							
3	15(45.45%)	0(0%)	3(5.56%)	3(4.41%)	0(0%)	/	18(16.22%)
4A	15(45.45%)	2(14.29%)	5(9.26)	7(10.29%)	0(0%)	/	22(19.82%)
4B	3(9.09%)	4(28.57%)	9(16,67%)	13(19.12%)	2(20.00%)	/	18(16.22%)
4C	0(0%)	3(21.43%)	9(16.67%)	12(17.65%)	2(20.00%)	/	14(12.60%)
5	0(0%)	5(35.71%)	23(42.59%)	28(41.18%)	1(10.00%)	/	29(26.13%)
6	0(0%)	0(0%)	2(3.70%)	2(2.94%)	0(0%)	/	2(1.80%)
NA	0(0%)	0(0%)	3(5.56%)	3(4.41%)	5(50.00%)	/	8(7.21%)

**Table 3:** Clinicopathologic characteristics of patients with malignant tumor.

	Malignancy (N=68)		
	Mastectomy (N=14)	Mastectomy and Immediate Breast Conserving (N=54)	All Malignancy (N=68)
Clinical stage			
DCIS	2(14.29%)	4(7.41%)	6(8.82%)
I	5(35.71%)	18(33.33%)	23(33.82%)
IIa	6(42.86%)	17(31.48%)	23(33.82%)
IIb	0(0%)	10(18.52%)	10(14.71%)
IIIa	0(0%)	1(1.85%)	1(1.47%)
NA	1(7.14%)	4(7.41%)	5(7.35%)
Histological grade			
I	0(0%)	6(11.11%)	6(8.82%)
II	9(64.29%)	36(66.67%)	45(66.18%)
III	2(14.29%)	8(14.81%)	10(14.71%)
NA	3(21.43%)	4(7.41%)	7(10.29%)
Lymph node metastasis			
Yes	3(21.43%)	17(31.48%)	20(29.41%)
No	11(78.57%)	35(64.82%)	46(67.65%)
NA	0(0%)	2(3.70%)	2(2.94%)
Adjuvant therapy			
Chemotherapy			
Yes	11(78.57%)	38(70.37%)	49(72.06%)
No	3(21.43%)	16(29.63%)	19(27.94%)
HER-2 targeted therapy			
Yes	5(35.71%)	13(24.07%)	18(26.47%)
No	9(64.29%)	41(75.93%)	50(73.53%)
Hormone therapy			
Yes	10(71.43%)	40(74.07%)	50(73.53%)
No	4(28.57%)	14(25.93%)	18(26.47%)
Immunotherapy			
Yes	3(21.43%)	10(18.52%)	13(19.12%)
No	11(78.57%)	44(81.48%)	55(80.88%)
Subtype			
Luminal A	3(21.43%)	16(29.63%)	19(27.94%)
Luminal B	7(50.00%)	21(38.89%)	28(41.18%)
HER-2 positive	1(7.14%)	8(14.81%)	9(13.24%)
Triple-negative	2(14.29%)	5(9.26%)	7(10.29%)
NA	1(7.14%)	4(7.41%)	5(7.35%)
ER			
Positive	9(64.28%)	38(70.37%)	47(69.12%)
Negative	3(21.43%)	12(22.22%)	15(22.06%)
NA	2(14.29%)	4(7.41%)	6(8.82%)
PR			
Positive	6(42.86%)	31(57.41%)	37(54.42%)
Negative	6(42.86%)	19(35.18%)	25(36.76%)
NA	2(14.29%)	4(7.41%)	6(8.82%)
HER-2			
Overexpressed	4(28.57%)	11(20.37%)	15(22.06%)
Negative	8(57.14%)	38(70.37%)	46(67.65%)
NA	2(14.29%)	5(9.26%)	7(10.29%)
Ki-67			
>14%	11(78.57%)	37(68.52%)	48(70.59%)
<14%	1(7.14%)	13(24.07%)	14(20.59%)
NA	2(14.29%)	4(7.41%)	6(8.82%)
Reconstruction type (N=54)			
Prosthesis	/	40(74.07%)	/
Expander	/	11(20.37%)	/
TRAM	/	3(5.56%)	/

The mean age of all patients was 36.97 ± 11.737 (11-67) years. Among the female patients, 84 (75.68%) had previous reproductive history. And only 3 (2.70%) of them were postmenopausal, the other 108 (97.30%) female patients were premenopausal. Among all of them, 25 patients (21.74%) had previous breast surgery. According to the analysis of the distribution of lesions in all patients, it was found that 59 (51.30%) patients had left-sided lesions, 51 (44.35%) patients had right-sided lesions, and the remaining 5 (4.35%) patients with bilateral lesions throughout the study. The mean maximum mass diameter of all patients was 3.00 ± 1.644 (0.6-8.5) cm. BI-RADS classification: 18 cases (16.22%) in grade 3, 22 cases (19.82%) in grade 4A, 18 cases (16.22%) in grade 4B, 14 cases (12.60%) in grade 4C, 29 cases (26.13%) in grade 5, and 2 cases (1.80%) in grade 6.

The age of benign lesion group was 25.94 ± 11.034 (11–60) years old, of whom 12 (36.36%) had a history of pregnancy and childbirth, and all this group patients were premenopausal. Also, there were three cases with a history of previous breast surgery, all of which were adenofibroma resections. Besides, in the benign lesion group, there were 15 left-sided lesions, 15 lesions on the right side (45.45%), and the other three lesions were bilateral (9.09%). The maximum diameter of the resected lesions in the benign lesion group was 4.13 ± 1.731 (1.3-8.5) cm on average. Preoperative B-ultrasound BI-RADS classification showed that 15 (45.45%) were grade 3, 15 (45.45%) were grade 4A, and the remaining 3 (9.09%) were grade 4B. The average intraoperative bleeding was 37.27 ± 16.564 (20–80) ml. The operation time was 80.61 ± 42.964 (30-250) minutes, and the length of hospital stay was 3.79 ± 1.066 (2–7) days. The average length of the incision was 3.83 ± 0.804 (3-5) cm. The average distance between the lesions and the NAC was 3.50 ± 1.044 (1–5) cm in 27 (81.82%) patients, and this data was missing in 6 (18.18%) patients.

The average age of malignant lesion group was 41.68 ± 7.120 (27–67) years old, 65 (95.59%) had a history of pregnancy, 66 (97.06%) were premenopausal, and 2 (2.94%) were postmenopausal. 11 (16.18%) patients had a history of breast surgery, including 7 patients who underwent breast mass resection, 2 patients who underwent breast adenofibroma resection, 1 patient who underwent tumor resection, and the last one was accepted a modified radical mastectomy. Among the 68 patients, 40 (58.82%) had left lesions, and the remaining 28 (41.18%) had right lesions. There were no bilateral lesions in this group. The maximum mass diameter was 2.42 ± 1.182 (0.6-5.8) cm. Preoperative B-ultrasound BI-RADS classification showed that 3 (4.41%) were grade 3, 7 (10.29%) were grade 4A, 13 (19.12%) were grade 4B, 12 (17.65%) were grade 4C, 28 (41.18%) were grade 5, and 2 (2.94%) were grade 6. The other three (4.41%) were not recorded. The average intraoperative bleeding in this group was 106.06 ± 41.227 (10–200) ml. The average operation time was 154.89 ± 46.774 (55-275) minutes, and the length of hospital stay was 5.69 ± 2.463 (2-18) days. The incision length was 3.70 ± 0.851 (3-8) cm. The distance between the tumor and the NAC in 61 (89.71%) patients was 3.41 ± 1.430 (0.5-7) cm. We also analyzed the clinical staging, pathological staging, molecular typing, and postoperative adjuvant therapy of 68 malignant tumor cases. In addition to the 6 cases (8.82%) of ductal carcinoma in situ, there were 23 cases (33.82%) of clinical stage I and clinical stage IIA, 10 cases (14.71%) of clinical stage IIB, 1 case (1.47%) of clinical stage IIIA, and the remaining 5 cases (7.35%) of clinical stage unknown. According to the biopsy results after surgery, there were 6 cases (8.82%) with histological grade I, 45 cases (66.18%) with histological grade II, 10 cases (14.71%) with artificial histological grade III, and 7 cases (10.29%) with a definite histological grade. Twenty (29.41%) patients were certified to have axillary lymph node metastasis, 46 (67.65%) patients were confirmed to have no axillary lymph node metastasis, but 2 (2.94%) patients could not be confirmed to have axillary lymph node metastasis. Sixty-eight cases of E-NSM were treated with postoperative adjuvant therapy, 49 cases (72.06%) received chemotherapy, 18 cases (26.47%) received anti-human epidermal growth factor receptor 2 (HER2) targeted therapy, 50 cases (73.53%) received endocrine therapy, and only 13 cases (19.12%) received immunotherapy. According to the immunohistochemical analysis, there were 19 cases (27.94%) of Luminal A type breast cancer, 28 cases (41.18%) of Luminal B type breast cancer, 9 cases (13.24%) of HER2 overexpression type, and 7 cases (10.29%) of triple-negative breast cancer. Immediate breast reconstruction after mastectomy was performed in 54 patients, of whom 11 (20.37%) were treated with dilator implant reconstruction and 40 (74.07%) were treated with silicone prostheses. And three (5.56%) patients used transverse rectus abdominal musculocutaneous (TRAM).

Among the special surgery group, 7 cases were after previous prosthesis implantation or breast augmentation, 2 cases were expander replacement prostheses, and 1 case was second-stage prosthesis implantation reconstruction after radical mastectomy. The average age of the patients was 47.40 ± 8.732 (33-66) years old, 7 (70.00%) patients had a history of pregnancy, 9 (90.00%) patients were premenopausal, and 1 (10.00%) patient was postmenopausal. Only 1 case (10.00%) had left lesions, 7 cases (70.00%) had right lesions, and the other 2 cases (20.00%) had bilateral lesions. The average intraoperative blood loss was 137.50 ± 54.486 (50-200) ml. The average operation time was 181.00 ± 62.042 (95–280) minutes, and the average length of hospital stay was 5.67 ± 2.749 (2–12) days. The average incision length was 4.80 ± 1.691 (3.5-8) cm. Due to the special type of surgery, only 3 patients had tumor sizes recorded. The average tumor size was 4.53 ± 1.808 (2.0-6.1) cm. The average distance from the NAC was 4.00 ± 1.414 (2-5) cm.

The average age of the 4 male patients was 22.00 ± 5.244 (15-28) years old, and only one patient had a history of breast surgery. Three cases (75.00%) had left lesions, and the other one (25.00%) had right lesions. The average breast thickness was 1.38 ± 0.540 (0.8-2.1) cm. The average intraoperative blood loss was 47.50 ± 32.692 (20–100) ml. The average operation time was 75.75 ± 15.990 (61–102) minutes, and the average length of hospital stay was 4.50 ± 0.433 (4-5) days. The average incision length was 3.63 ± 0.820 (3-5) cm.

In the follow-up of tumor prognosis and oncological safety, only one patient had a positive surgical margin, and extended resection was performed 1 month after the first operation. As of November 30, 2022, no patient had experienced local recurrence or distant metastasis.

Patients included in the study were followed up to assess postoperative cosmetic outcomes, as detailed in **Table 4**. Patients were 100% satisfied with the appearance of the breast after dressing, among whom 23.48% were very satisfied with the postoperative breast appearance. 57.39% of patients were satisfied with the appearance of the breast in the nudist state after surgery, and 18.26% were very satisfied. Meantime, we also carried out a follow-up on the satisfaction of postoperative breast size. In addition to the 23.48% of the patients who expressed general satisfaction, the remaining 76.52% of the patients were satisfied, among whom 20.00% were very satisfied with the postoperative breast size. Regarding the common concern about postoperative bilateral breast symmetry, the follow-up results showed that 64.35% of patients were satisfied with bilateral breast symmetry after E-NSM, while the satisfaction rate for bilateral nipple and areola location was as high as 90.43%. Another problem affecting the long-term aesthetic effect after surgery is the surgical incision. In the follow-up, we found that 94.78% of the 115 patients were satisfied with the location of their incision, and 86.96% were very satisfied with the incision length. In addition, all patients had no wound suppuration, wound dehiscence, or poor wound healing occurred in the wound healing of 115 patients, and 94.78% of the patients were satisfied with the wound healing state. According to the feedback received during the follow-up, one patient had mild prosthetic incompatibility and postoperative chest pain, one patient had nipple and areola blackness, and one patient had suture malabsorption. One patient developed postoperative drainage obstruction complicated by infection and recovered after active anti-inflammatory conservative treatment. Another patient had bleeding, a short-term postoperative complication, without other serious postoperative complications.

**Table 4:** Operation related indicators.

	Benign(N=33)	Malignancy (N=68)			Special Type(N=10)	Male Breast Cancer(N=4)	All patients(N=115)
		Mastectomy(N=14)	Mastectomy and Immediate Breast Conserving (N=54)	All Malignancy(N=68)			
**Blood loss (ml)**	37.27 ± 16.564 (20-80)	85.00 ± 33.112 (10-150)	111.73 ± 41.358 (50-200)	106.06 ± 41.227 (10-200)	137.50 ± 54.486 (50-200)	47.50 ± 32.692 (20-100)	85.77 ± 50.342 (10-200)
**OP time (min)**	80.61 ± 42.964 (30-250)	140.71 ± 52.978 (55-230)	158.71 ± 44.187 (85-275)	154.89 ± 46.774 (55-275)	181.00 ± 62.042 (95-280)	75.75 ± 15.990 (61-102)	131.84 ± 59.332 (30-280)
**Hospital stay (days)**	3.79 ± 1.066 (2-7)	6.21 ± 3.745 (2-18)	5.55 ± 1.967 (3-15)	5.69 ± 2.463 (2-18)	5.67 ± 2.749 (2-12)	4.50 ± 0.433 (4-5)	5.05 ± 2.305 (2-18)
**Length of incision (cm)**	3.83 ± 0.804 (3-5)	3.70 ± 0.245 (3.5-4)	3.74 ± 0.848 (3-8)	3.70 ± 0.851 (3-8)	4.80 ± 1.691 (3.5-8)	3.63 ± 0.820 (3-5)	3.83 ± 0.884 (3-8)
Distance of mass from nipple(cm)							
Definite	27(81.82%)/3.50 ± 1.044 (1-5)	12(85.71%)/3.42 ± 1.320 (2-7)	49(90.74%)/3.41 ± 1.457 (0.5-7)	61(89.71%)/3.41 ± 1.430 (0.5-7)	3(30.00%)/4.00 ± 1.414 (2-5)	/	91(79.13%)/3.46 ± 1.337 (0.5-7)
NA	6(18.18%)	2(14.29%)	5(9.26%)	7(10.29%)	7(70.00%)	/	24(20.87%)

## Discussion

With the innovation of medical technology, the application of endoscopic surgery covers almost all areas of surgery. From its beginnings as laparoscopy and thoracoscopy in the natural body cavity to potential applications in the peritoneal, thyroid and joint cavities between cavities or closures, the use and development of endoscopic surgery is a natural consequence of the trend of the times. As early as 1992, endoscopic surgery was first used to remove contractures after plastic surgery. In 1995, endoscopy was used for the first time for total mastectomy and the treatment of benign breast tumors. Up until now, mammary endoscopic technology has been widely used in breast surgery such as axillary lymph node dissection, breast conservation, subcutaneous mastectomy and breast reconstruction with implants (2, 3, 7, 9, 21, 23–26). We reported 115 cases of endoscopic surgery assisted by the transaxillary single port insufflation technique, in which a single axillary incision was the main incision. According to the results of our preliminary analysis, we confirmed the feasibility of transaxillary single port insufflation technique-assisted endoscopic surgery, i.e., its acceptable safety and aesthetics in breast disease surgery. In an era when surgical treatments of breast tumors and other breast diseases is increasingly emphasising precision, minimally invasive, and functional preservation, we believe that this technique will continue to be refined and become increasingly widely used.

In breast surgery, the traditional surgical approach leaves one or even more visible scars, which not only seriously affect the aesthetics of the breast, but also causes great trauma to the patient’s heart and could have a negative emotional impact after the operation (18, 27). Moreover, the surgical incision on the surface of the breast is subjected to tension from the implant after surgery, which may lead to widening of the scar, incision dehiscence and exposure of the implant. Therefore, in traditional one-stage breast reconstruction surgery, patches and titanium mesh are often chosen to cover the surface of the implant in order to avoid complications such as implant exposure, which can result in damage to the implant or increased costs and increased financial stress for the patient (1–3, 5, 6). The use of endoscopic techniques in breast surgery has brought a turnaround to these problems. Endoscopic surgery means visualizing the excision, allowing for a more delicate operation. All breast tissue is removed while preserving the nipple and areola, and the preservation of the subcutaneous fat layer of the breast protects the subcutaneous blood flow to the breast, reducing the incidence of subcutaneous necrosis.

The traditional transaxillary incision technique-assisted endoscopy usually consists of two or three incisions, including an incision under the axilla and a small incision around the NAC. To reduce the risk of NAC ischemia or necrosis, some studies suggest that the length of the incision around the areola should be limited to 1/3 of a circle during E-NSM. However, even with such a small incision, ischemic and necrosis of the NAC may still occur in some patients who are prone to ischemia or necrosis, including elderly patients, women with breast enlargement or prolapse, and patients with a history of smoking or previous radiation exposure (28). To solve such problems, Du and his team (29) creatively created Huaxi No.1 foramen around the areola to reduce the possibility of ischemic necrosis of NAC and the difficulty of the operation.

In order to expose the surgical field and expand the operating space, traditional endoscopic techniques use carbon dioxide perfusion combined with lipolysis or suspension combined with lipolysis (24). Lipolysis is performed with the aid of a lipolysis solution, which preserves the reticular tissue fibers and lymph node structures of the axilla or breast after liposuction, with clear and easily accessible cavities. The suspension approach creates an operative space by suspending the skin over the surgical area, thus avoiding compression of the tumor and reducing the risk of tumor spread. However, the safety concerns associated with lipolysis are controversial. Some studies have concluded that lipolysis and liposuction operations are contrary to the principles of complete tumor excision and tumor free (30, 31). The suspension approach creates limited space, and the suspension instruments inevitably cause additional damage to the skin of the breast affecting the postoperative recovery (32). Breast endoscopic surgery itself has its own and common postoperative complications, such as subcutaneous emphysema formation, the development of hypercapnia, intraoperative and postoperative bleeding, important neurovascular injuries, and tumor recurrence. To overcome or improve these problems, the techniques of breast endoscopic surgery were progressively advancing. The transaxillary single-incision inflation technique assisted endoscopic surgery reported in this study is an improved surgical approach that may address some of these problems. In our study, no NAC ischemia or necrosis occurred, and all patients had no local recurrence or distant metastases at 1 year postoperatively. This suggested that the oncological safety of single-incision endoscopic insufflation technique assisted transaxillary surgery is reliable at short-term follow-up. It is easy to see from the patient-centered postoperative follow-up results that the patients were generally with the postoperative aesthetic outcome of this surgical technique.

## Conclusion

Based on the results of this study, we believe that transaxillary single incision insufflation technique-assisted endoscopic surgery has great potential for patients with benign breast disease or early-stage breast cancer, negative axillary lymph nodes, appropriate breast size (below C cup), and non-severe breast ptosis. The endoscopic technique allows for clearer dissection, resulting in less blood loss during surgery, the identification and preservation of important axillary vascular and nerve structures, and better post-operative aesthetic results, including concealed incisions, small wounds, beautiful appearance and high patient satisfaction. However, the indications for transaxillary single incision insufflation technique-assisted endoscopic surgery are limited to patients with large breasts and severe breast ptosis, so there is still room and necessity for improvement in this technique. Therefore, the development of breast endoscopic technology still needs more practical theoretical research support. We should take a critical view of the development of breast endoscopic surgery. The safety of the tumor should not be neglected in the mere pursuit of postoperative aesthetics, striving to provide the least surgical incisional damage and the best postoperative appearance for patients with breast disease in the safest possible way.

## Data availability statement

The original contributions presented in the study are included in the article/supplementary material. Further inquiries can be directed to the corresponding author.

## Ethics statement

Ethical review and approval was not required for the study on human participants in accordance with the local legislation and institutional requirements. The patients/participants provided their written informed consent to participate in this study.

## Author contributions

XFW, XW and LL participated in design, the analysis and interpretation of the data and drafted manuscript. XL, RM and XS collected the data and flowed up. CX revised and approved the final version of the manuscript. All authors contributed to the article and approved the submitted version.
